# Crushed Gold Shell Nanoparticles Labeled with Radioactive Iodine as a Theranostic Nanoplatform for Macrophage-Mediated Photothermal Therapy

**DOI:** 10.1007/s40820-019-0266-0

**Published:** 2019-05-03

**Authors:** Sang Bong Lee, Jae-Eon Lee, Sung Jin Cho, Jungwook Chin, Sang Kyoon Kim, In-Kyu Lee, Sang-Woo Lee, Jaetae Lee, Yong Hyun Jeon

**Affiliations:** 1 New Drug Development Center, Daegu-Gyeongbuk Medical Innovation Foundation, Daegu, South Korea; 2 Department of Biomaterials Science, College of Natural Resources and Life Science/Life and Industry Convergence Research Institute, Pusan National University, Pusan, South Korea; 3 Leading-Edge Research Center for Drug Discovery and Development for Diabetes and Metabolic Disease, Kyungpook National University Hospital, Daegu, 702-210 South Korea; 4 Department of Nuclear Medicine, Kyungpook National University Hospital, Daegu, 702-210 South Korea; 5 Department of Internal Medicine, Kyungpook National University School of Medicine, Daegu, 700-721 South Korea; 6 Daegu-Gyeongbuk Medical Innovation Foundation, Daegu, 360-4 South Korea; 7 Laboratory Animal Center, Daegu-Gyeongbuk Medical Innovation Foundation, Daegu, 360-4 South Korea

**Keywords:** Photothermal therapy, Radionuclide crushed gold shell nanoballs, Macrophage-mediated delivery

## Abstract

Plasmonic nanostructure-mediated photothermal therapy (PTT) has proven to be a promising approach for cancer treatment, and new approaches for its effective delivery to tumor lesions are currently being developed. This study aimed to assess macrophage-mediated delivery of PTT using radioiodine-124-labeled gold nanoparticles with crushed gold shells (^124^I-Au@AuCBs) as a theranostic nanoplatform. ^124^I-Au@AuCBs exhibited effective photothermal conversion effects both in vitro and in vivo and were efficiently taken up by macrophages without cytotoxicity. After the administration of ^124^I-Au@AuCB-labeled macrophages to colon tumors, intensive signals were observed at tumor lesions, and subsequent in vivo PTT with laser irradiation yielded potent antitumor effects. The results indicate the considerable potential of ^124^I-Au@AuCBs as novel theranostic nanomaterials and the prominent advantages of macrophage-mediated cellular therapies in treating cancer and other diseases.

**Electronic supplementary material:**

The online version of this article (10.1007/s40820-019-0266-0) contains supplementary material, which is available to authorized users.

## Highlights

- Crushed gold shell radionuclide nanoballs (^124^I-Au@AuCBs) were fabricated as unique photothermal therapeutics and nuclear medicine imaging nanoplatforms.
- Macrophage-mediated delivery of ^124^I-Au@AuCBs and their potential photothermal therapy applications were demonstrated in mice with colon cancer.

**Electronic supplementary material:**

The online version of this article (10.1007/s40820-019-0266-0) contains supplementary material, which is available to authorized users.

## Graphical abstract

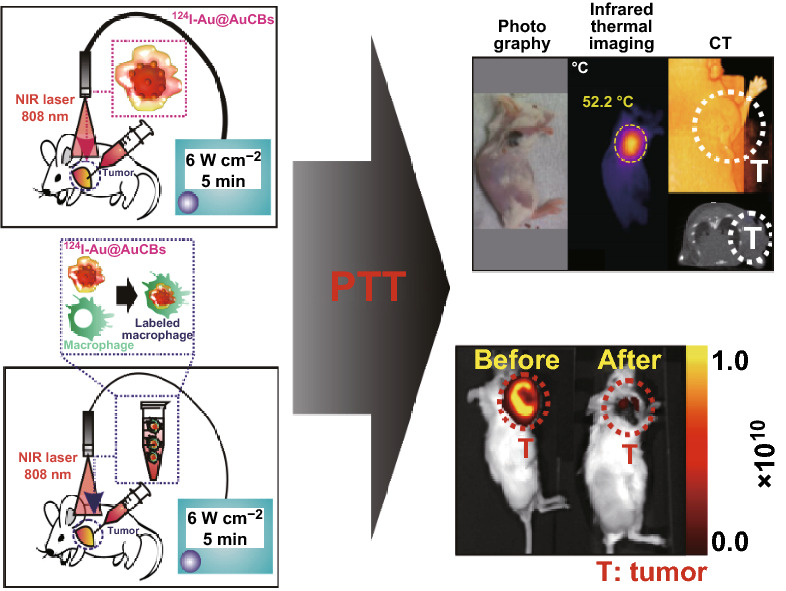

## Introduction

Gold nanomaterials have unique properties, such as strong localized surface plasmon resonance, biocompatibility, and easy surface modification via thiol–gold bonding [1, 2]. Numerous recent studies have attempted to develop several types of gold nanomaterials, such as Au nanoshells [3–6], Au nanocages [7–10], Au nanorods [1, 11–15], Au nanovesicles [16], Au nanostars [17–19], and Au nanohexapods [18], that are compatible with theranostic agents. In particular, plasmonic gold nanoparticles (AuNPs) have been widely studied as photothermal agents, owing to their excellent photothermal conversion induced by near-infrared (NIR) irradiation. AuNP-mediated photothermal therapy (PTT) has proven to be a promising treatment strategy for various cancers [19]. However, several issues remain to be resolved for successful cancer therapy. In particular, the efficient delivery of PTT-compatible AuNPs to entire tumor lesions is required to ensure a cell death-inducing temperature.

Several approaches have been adopted to effectively deliver photothermal agents to tumor lesions, via passive or active targeting [1, 20–26]. For passive targeting, via enhanced permeability retention (EPR) effects, several types of coating materials (e.g., polyethylene glycol polymer, chitosan) have been introduced to the surface of AuNPs. To actively target tumor lesions, tumor-specific antibodies or ligands have been adopted to functionalize the AuNPs. Although these approaches have yielded promising results in delivering photothermal agents to tumors, nanoparticles can be entrapped in organs capable of EPR, such as the liver, kidney, and spleen, thereby inducing severe toxicity in normal organs. As an alternative delivery approach, intratumoral injection is useful to treat breast cancer and melanoma, owing to the accurate targeted delivery of PTT agents. However, most injected particles are retained in tumor lesions proximal to the site of injection and cannot penetrate deep into the tumors, thereby resulting in poor therapeutic outcomes. Thus, new approaches should be investigated for the effective delivery of photothermal agents to tumor lesions to elicit drastic therapeutic responses and to reduce toxicity in vital organs.

Macrophages are suitable transporters of various nanoparticles because they are present in the circulation and are easy to harvest from patients. Furthermore, owing to their unique properties, they easily engulf nanoparticles, such that each cell behaves as a “Trojan horse” delivery system, enabling the infiltration of otherwise inaccessible tumor lesions. Furthermore, several studies have reported the successful macrophage-mediated delivery of theranostic biomaterials to tumor lesions and resulting therapeutic effects in glioma, liver, and lung cancer models [27–31], revealing its great potential for cancer treatment.

We recently developed highly stable, biocompatible, and sensitive radioiodine-labeled AuNPs with crushed gold shells (^124^I-Au@AuCBs) as positron emission tomography/computed tomography (PET/CT) imaging agents for in vivo tumor imaging [32] and suggested their possible use in various biological applications. However, despite interesting findings regarding ^124^I-Au@AuCBs as useful biomaterials, we have not yet investigated the possibility of using ^124^I-Au@AuCBs as PTT agents. The generation of effective photothermally converted biomaterials is facilitated by the nanogap between the gold core and the gold shell, thus transforming these nanomaterials into promising theranostic biomaterials.

**Fig. 1:**
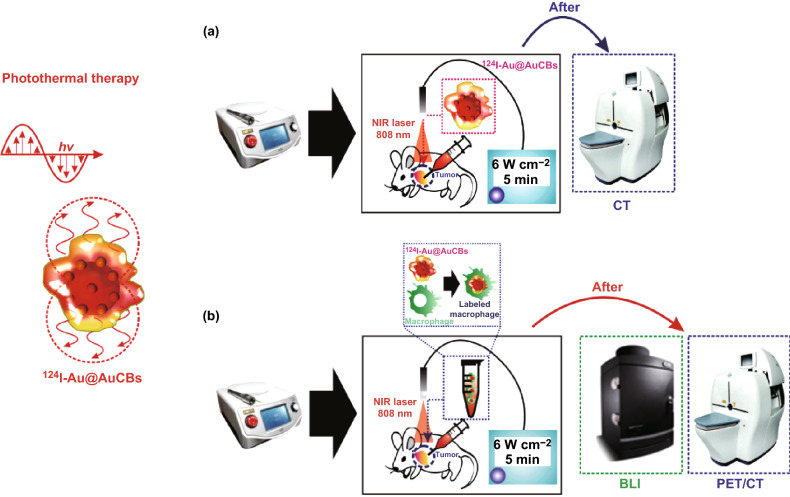
A schematic representation of the evaluation of **a** in vivo photothermal conversion effects of ^124^I-Au@AuCBs and **b** in vivo photothermal therapy using ^124^I-Au@AuCB-labeled macrophages in a colon cancer model, followed by positron emission tomographic imaging of ^124^I-Au@AuCB-labeled macrophage distribution in tumor lesions

This study aimed to investigate the feasibility of using ^124^I-Au@AuCBs as photothermal therapeutics and imaging nanoplatforms in mice with colon cancer (as shown in Fig. 1) and the possibility of macrophage-mediated delivery of ^124^I-Au@AuCBs for PTT applications in mice with colon cancer.

## Materials and Methods

### Materials and Instruments

All chemical reagents and tannic acid-capped AuNPs were purchased form Ted Pella, Inc. (Redding, CA, USA), and Na^124^I (half-life 4.2 days, emission type: high-energy γ and positron, energy 811 keV) was provided by KIRAMS (Seoul, South Korea). HAuCl_4_ was purchased from Sigma-Aldrich (St Louis, MO, USA).

PET/CT imaging was performed with a PET/CT scanner (LabPET8; Gamma Medica-Ideas, Waukesha, WI, USA). Bioluminescence imaging (BLI) was performed using an IVIS Lumina III instrument (PerkinElmer, Waltham, MA, USA). Photothermal images were obtained using a digital thermometer (TES Electrical Electronic Corp, Taipei, Taiwan) and an NIR imaging camera (FLIR Systems, Wilsonville, OR, USA).

### Animals and Cells

Specific pathogen-free immunocompetent 6-week-old BALB/c mice were obtained from SLC, Inc. (Shizuoka, Japan). All experimental procedures involving animals were performed in strict accordance with the appropriate institutional guidelines for animal research. This protocol was approved by the Committee on the Ethics of Animal Experiments of the Kyungpook National University (approval number: KNU 2012-43).

Murine colon cancer CT26 cells co-expressing firefly and mCherry genes (CT26/FM) were grown in RPMI medium 1640 supplemented with 10% fetal bovine serum and 1% penicillin–streptomycin (Gibco, Waltham, MA, USA).

Murine macrophage Raw264.7 cells were cultured in Dulbecco’s modified Eagle medium supplemented with 10% fetal bovine serum and 1% antibiotic–antimycotic (Invitrogen, Carlsbad, CA, USA) at 37 °C in a 5% CO_2_ atmosphere.

### Preparation of Crushed Gold Shell Radionuclide Nanoballs

We have previously reported ^124^I-Au@AuCBs synthesis methods [32]. Briefly, to generate a crushed gold shell on ^124^I-AuNPs, 1.0 mL of ^124^I-AuNPs (1 nM) was mixed with 500 μL of 1.0% (w/v) poly(*N*-vinyl-2-pyrrolidone) (MW, 40 kDa) and 100 μL of 100 mM phosphate buffer (pH 7.5 and 12.0). The solution was mixed with 434 μL of hydroxylamine hydrochloride (10 mM) and 434 μL of HAuCl_4_ (5 mM), gently vortexed for 30 min at room temperature, and centrifuged twice at 6500 rpm for 15 min. The resulting supernatant was resuspended in 1.0 mL of distilled water.

### Characterization of Crushed Gold Shell Radionuclide Nanoballs

UV–Vis spectroscopy was performed using a Cary 60 UV–Vis spectrometer (Agilent Technologies, Santa Clara, CA, USA). Transmission electron microscopy (TEM) and energy-dispersive X-ray (EDX) mapping were performed using an FEI Tecnai F20 transmission electron microscope (FEI Company, Eindhoven, the Netherlands). The hydrodynamic size of the nanoparticles was measured using ζ-potentials (ELS-Z, Otsuka, Japan). Fourier transform infrared (FT-IR) spectra were recorded on a PerkinElmer spectrometer in the range between 4000 and 400 cm^−1^.

### Photothermal Conversion In Vitro

The photothermal conversion was evaluated using an 808 nm NIR laser (LVI, Anyang, South Korea). ^124^I-Au@AuNPs and ^124^I-Au@AuCBs were suspended in glass vials (1 mL) at different concentrations (0.5, 1, 2, and 4 nM) and exposed to the NIR laser at different power settings (3, 6, and 9 W cm^−2^). The temperatures of the suspensions were recorded using a digital thermometer (TES Electrical Electronic Corp, Taipei, Taiwan), with an accuracy of ± 0.1 °C. To determine whether exposure to the NIR laser could damage the morphology of the gold nanomaterials, they were examined using TEM, before and after irradiation at 9 W cm^−2^ for 5 min.

### In Vitro Photothermal Therapy

The following experiments were performed to evaluate the photothermal therapeutic effects of ^124^I-Au@AuCBs on cancer cells.

#### Study 1

Either CT26 or CT26/FM cells were seeded in 96-well plates and incubated with ^124^I-Au@AuCBs (2 nM) for 3 h, followed by irradiation with an NIR laser (808 nm, 6 W cm^−2^). After 2 days, cell morphology was determined by microscopic imaging (Leica, Wetzlar, Germany). Cell proliferation was evaluated using a Cell Counting Kit (CCK-8; Dojindo Laboratories, Tokyo, Japan), and in vitro bioluminescent imaging was performed using an IVIS Lumina III instrument.

For cell proliferation assays, 10 µL of CCK-8 solution was added to treated cells and the absorbance was measured at 450 nm using a microplate reader (BMG Labtech, Offenburg, Germany).

#### Study 2

Either CT26 or CT26/FM cells were seeded in 96-well plates at 24 h and incubated with ^124^I-Au@AuCB (2 nM)-labeled macrophages, followed by exposure to an NIR laser (808 nm, 6 W cm^−2^) for 5 min. Two days later, cell viability was determined by in vitro bioluminescent imaging using an IVIS Lumina III instrument.

### Apoptosis Analysis

Treated cells were collected, stained with FITC-conjugated annexin V and propidium iodide (BD Biosciences, San Jose, CA, USA), and analyzed by flow cytometry using a BD Accuri C6 flow cytometer (BD Biosciences).

### In Vivo Photothermal Therapy

#### Study 1

CT26 cells were injected subcutaneously into mice, and tumor-bearing mice were divided into the following two groups when tumor mass was detected by physical inspection and palpation: Group 1, free ^124^I-injected group; Group 2, ^124^I-Au@AuCB-injected group. Tumor lesions were exposed to an NIR laser (808 nm, 6 W cm^−2^, 5-min exposure), and temperature changes in the tumor lesion were monitored using a digital thermometer. Nine days after therapy, the tumor was excised and weighed.

#### Study 2

CT26/FM cells were injected subcutaneously into mice, and tumor-bearing mice were divided into the following five groups when tumor volume reached 100–120 mm^3^: Group 1, phosphate-buffered saline (PBS)-treated group; Group 2, NIR laser-treated group; Group 3, ^124^I-Au@AuCB-loaded macrophage group; Group 4, ^124^I-Au@AuCB-loaded macrophage + NIR laser**-**treated group; and Group 5, unlabeled macrophage + NIR laser-treated group.

After injection of ^124^I-Au@AuCB-labeled macrophages into each tumor, PET/CT imaging was performed to determine the successful delivery of the particles to the tumor site. After image acquisition (3 h after intratumoral injection of ^124^I-Au@AuCB-labeled macrophages), the tumor lesion was exposed to an NIR laser (808 nm, 6 W cm^−2^, 5-min exposure) and temperature changes in the tumor lesion were monitored using a digital thermometer. Therapeutic response was evaluated by in vivo fluorescent imaging of the mCherry reporter gene. At day 9 after therapy, tumors were excised and weighed.

### PET/CT Imaging

For computed tomography imaging, a 20-min scan (tumor lesion imaging) was performed using a Triumph II PET/CT system (LabPET8; Gamma Medica-Ideas). For PET/CT imaging, a 15-min scan was performed using the same animal PET/CT system as described above. CT scans were performed with an X-ray detector (fly acquisition; number of projections 512; binning setting 2 × 2; frame number 1; X-ray tube voltage 75 kVp; focal spot size 50 μm; magnification factor 1.5; matrix size 512). CT images were reconstructed using filtered back-projections. All mice were anesthetized using 1–2% isoflurane gas during imaging. CT images were reconstructed using the 3D image visualization and analysis software, VIVID (Gamma Medica-Ideas).

### In Vivo Fluorescent Imaging

For CT26/FM tumor imaging, in vivo fluorescent imaging (FLI) was performed at the indicated times after PTT, using an IVIS Lumina III instrument with filter settings for mCherry. Grayscale photographic images and fluorescent color images were superimposed using LIVING IMAGE (version 2.12, PerkinElmer) and IGOR Image Analysis FX software (WaveMetrics, Lake Oswego, OR, USA). FLI signals were expressed in units of photon per cm^2^ per second per steradian (P cm^−2^ s^−1^ sr^−1^).

### Statistical Analysis

All data are expressed as the mean ± standard deviation (SD) from at least three representative experiments, and statistical significance was determined by unpaired Student’s tests using Prism 5 software (GraphPad, San Diego, CA, USA). Differences with *P* values less than 0.05 were considered statistically significant.

## Results and Discussion

### Characterization of ^124^I-Au@AuCBs as Photothermal Agents

We recently developed highly sensitive and stable PET/CT imaging agents, comprising a radioiodine-labeled gold core and a crushed gold shell (^124^I-Au@AuCBs) [32] or round gold shell (^124^I-Au@AuNPs) [33]. Several studies have reported the importance of morphological modification of nanomaterials and intra-nanogaps for the induction of photothermal conversion. ^124^I-Au@AuCBs and ^124^I-Au@AuNPs exhibit a crushed or round gold shell shape, respectively, with an intra-nanogap of 0.21–0.25 nm. These results led us to further examine the possibility of their use as new photothermal nanomaterials.

**Fig. 2:**
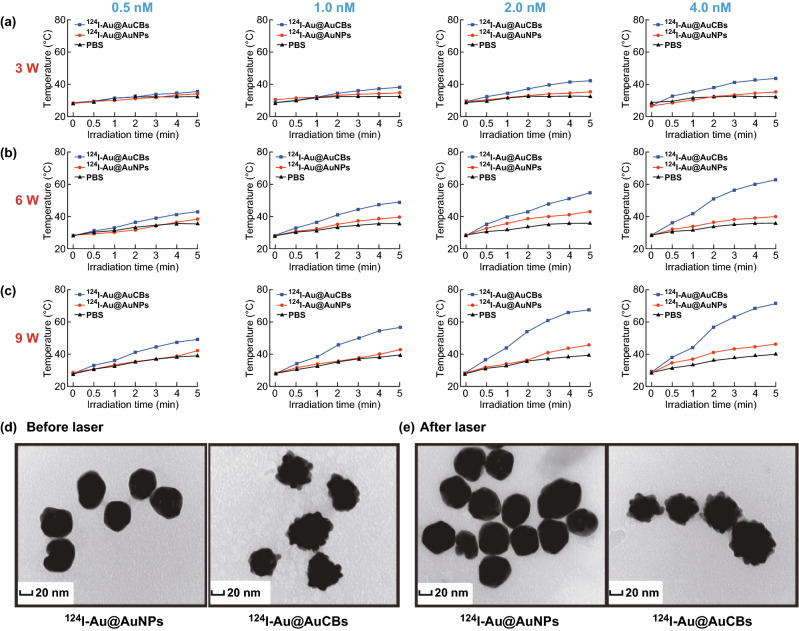
Photothermal conversion properties of ^124^I-Au@AuCBs. **a**–**c** Temperature change of PBS, ^124^I-Au@AuNP, and ^124^I-Au@AuCB solutions after irradiation with an NIR laser for 5 min at varying power levels (3, 6, and 9 W cm^−2^) and particle concentrations (0.5, 1.0, 2.0, and 4.0 nM). HR-TEM images of **d** ^124^I-Au@AuNPs and **e** ^124^I-Au@AuCBs, before and after irradiation (9 W cm^−2^, 5 min)

First, we investigated the localized surface plasmon resonance (LSPR) peaks of our imaging agents. The LSPR peaks were approximately 520 and 600 nm for the ^124^I-Au@AuNPs and ^124^I-Au@AuCBs, respectively (Fig. S1). Furthermore, we determined the effect of photothermal conversion of ^124^I-Au@AuNPs and ^124^I-Au@AuCBs after irradiation with an 808 nm NIR laser in micro-glass vials. As shown in Fig. 2a–c, NIR laser beam (3, 6, and 9 W cm^−2^) energy-dependent and AuNP (0.5, 1.0, 2.0, and 4.0 nM) concentration-dependent photothermal conversion effects were observed in ^124^I-Au@AuCB solutions, but not in PBS or ^124^I-Au@AuNP solutions. To investigate whether heating of the NIR laser influenced the nanostructure of ^124^I-Au@AuCBs, high-resolution transmission electron microscopic (HR-TEM) examination of the gold nanoplatform was performed, before and after irradiation. When compared with its structure prior to irradiation, no obvious morphological changes were detected after irradiation (Fig. 2d, e). These results suggest that the unique structure of ^124^I-Au@AuCBs could undergo photothermal conversion, with excellent stability, during PTT.

In addition, we investigated various characteristics of ^124^I-Au@AuCBs. The ^124^I-Au@AuCBs had bumpy surfaces, as evident from HR-TEM results. Subsequently, Au and I distributions were determined around the AuCBs by X-ray energy distribution mapping analysis (Fig. S2). Zeta-potential analysis results revealed that the surface charges of the particles were − 44.73 ± 2.18, − 50.73 ± 5.49, and − 31.66 ± 3.2 mV for AuNPs, ^124^I-AuNPs, and ^124^I-Au@AuCBs, respectively (Fig. S3a). FT-IR spectroscopy analysis revealed peaks at 1213 cm^−1^ for CH_2_, 1651 cm^−1^ for C=O stretching, 2858 cm^−1^ for C–H stretching, and 3408 cm^−1^ for O–H stretching. The FT-IR spectrum of AuCB products was the same as the spectra of the products of chemical reactions of their respective gold nanomaterials (Fig. S3b).

### Photothermal Therapeutic Effect of ^124^I-Au@AuCBs on Colon Cancer In Vitro and In Vivo

**Fig. 3:**
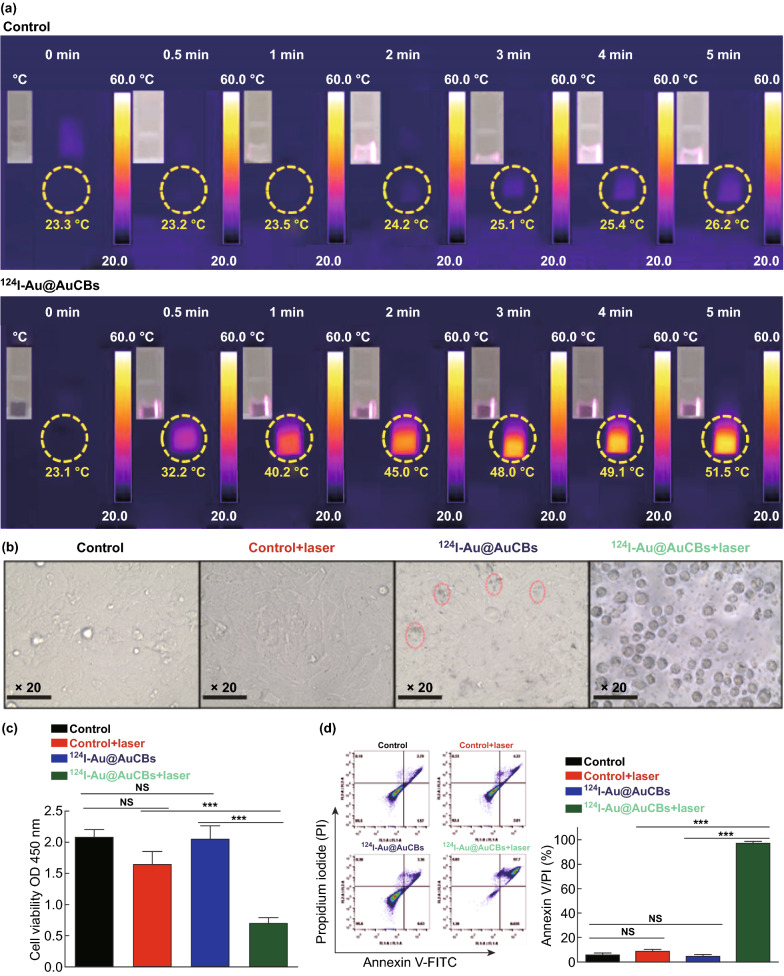
Cytotoxic effects of ^124^I-Au@AuCBs in colon cancer CT26 cells after photothermal therapy. **a** NIR images of ^124^I-Au@AuCB-labeled CT26 cells after irradiation with an NIR laser (6 W cm^−2^, 5 min). **b** Microscopic images of ^124^I-Au@AuCB-labeled or ^124^I-Au@AuCB-labeled CT26 cells irradiated with an NIR laser. **c** Analysis of cell viability and **d** apoptosis in irradiated cells via annexin V/PI staining. ***P < 0.0001; *NS* not significant

The photothermal therapeutic effect of ^124^I-Au@ AuCBs was determined by a cell proliferation assay. First, to investigate the adverse effects of ^124^I-Au@AuCBs on the proliferation of colon cancer cells, cells were cultured with ^124^I-Au@AuCBs at various concentrations for 24 h. As shown in Fig. S4a, cell viability remained unchanged in the unlabeled and labeled groups at the tested doses, indicating the absence of cytotoxicity. Furthermore, to demonstrate the photothermal therapeutic effects of ^124^I-Au@AuCBs on colon cancer CT26 cells, cells were incubated with ^124^I-Au@AuCBs (2 nM, 3 h) and then irradiated with a laser beam (6 W cm^−2^, 5 min), accompanied by photothermal imaging. Irradiated cells were seeded in 96-well plates and further incubated (additional 12 h) to evaluate PTT-mediated cytotoxic effects. As shown in Figs. 3a and S4b, a rapid temporary increase in temperature was observed immediately after irradiation. The CCK-8 assay results (Fig. 3c) showed a marked reduction in cell proliferation in irradiated and ^124^I-Au@AuCB-labeled cells, but not in non-irradiated or ^124^I-Au@AuCB-labeled cells, which concurred with the results of microscopic analysis (Fig. 3b) and annexin V/PI staining (Fig. 3d). These observations indicate that ^124^I-Au@AuCBs induced in vitro PTT-mediated cytotoxic effects via effective photothermal conversion.

**Fig. 4:**
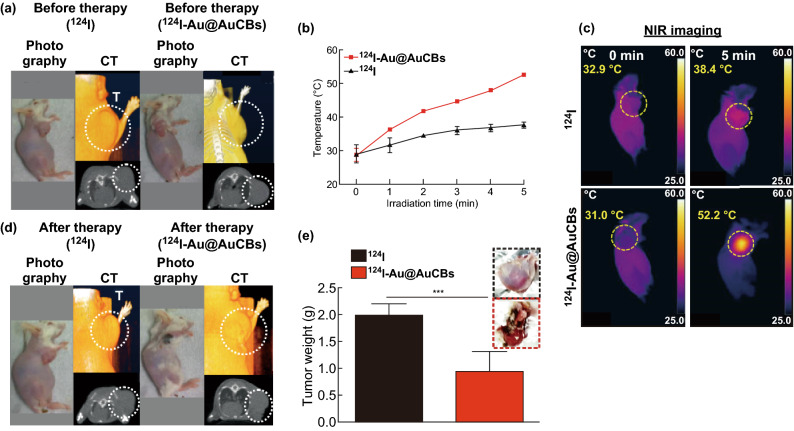
Evaluation of the effects of photothermal therapy with ^124^I-Au@AuCBs in CT26 tumor-bearing mice. **a**, **d** Photograph and computed tomographic imaging of CT26 tumor-bearing mice, before and after PTT with ^124^I-Au@AuCBs. CT26 tumor-bearing mice were administered either ^124^I or ^124^I-Au@AuCBs, and tumors were exposed to an NIR laser irradiation. **b** Temperature changes in either free ^124^I-or ^124^I-Au@AuCB-injected CT26 tumors after irradiation with an NIR laser (9 W cm^−2^, 5 min). **c** NIR imaging of CT26 tumor-bearing mice, before and after irradiation with an NIR laser. **e** Measurement of tumor weight after photothermal therapy. Inset: upper, CT26 tumors with ^124^I and NIR laser treatment; bottom, CT26 tumors with ^124^I-Au@AuCBs and NIR laser treatment. In vivo experiments were performed in duplicate (*n* = 7 mice per group). ***P < 0.0001

To determine the photothermal therapeutic effects of ^124^I-Au@AuCBs in vivo, CT26 tumor-bearing mice were grouped as follows: Group 1, free ^124^I + laser; Group 2, ^124^I-Au@AuCBs (injected doses 2 nM per 100 μL) + laser. Twenty minutes after the intratumoral injection of ^124^I-Au@AuCBs, tumor lesions were exposed to the laser (6 W cm^−2^, 808 nm, Fig. S5). Infrared thermal imaging was performed to monitor temperature changes in the tumor lesions during PTT. As shown in Fig. 4b, c, the temperature of the ^124^I-Au@AuCB-injected tumor increased with increasing irradiation time. After irradiation for 5 min, the temperatures were 31 and 52.2 °C in the free ^124^I-injected tumor and the ^124^I-Au@AuCB-injected tumor, respectively. Three minutes after irradiation, the temperature increased beyond 43 °C, which is known to damage cancer cells, owing to their relatively low heat tolerance resulting from a poor blood supply [34]. After NIR laser irradiation, the tumor-bearing mice were maintained and PTT-therapeutic outcomes were evaluated 9 days post-treatment, via both CT imaging and tumor weight measurement. CT imaging (Fig. 4a, d) showed a marked reduction in tumor mass in mice treated with ^124^I-Au@AuCBs + laser, which concurred with the results of tumor weight measurements (Fig. 4e). These data indicate that ^124^I-Au@AuCBs have unique properties to induce PTT-mediated cytotoxic effects via effective photothermal conversion in vitro and in vivo.

### Effects of PTT with ^124^I-Au@AuCB-Labeled Macrophages on Colon Cancer In Vitro and In Vivo

The development of nanoparticle systems with a high uptake efficiency is vital for successful cell-mediated photothermal therapy. Macrophages can be easily labeled with nanoparticles owing to their unique phagocytic activity [35]. Thus, several studies have attempted to load therapeutic or imaging particles into macrophages for cell tracking and effective in vivo delivery of cytotoxic agents (doxorubicin) throughout tumor lesions [29, 31, 36, 37].

**Fig. 5:**
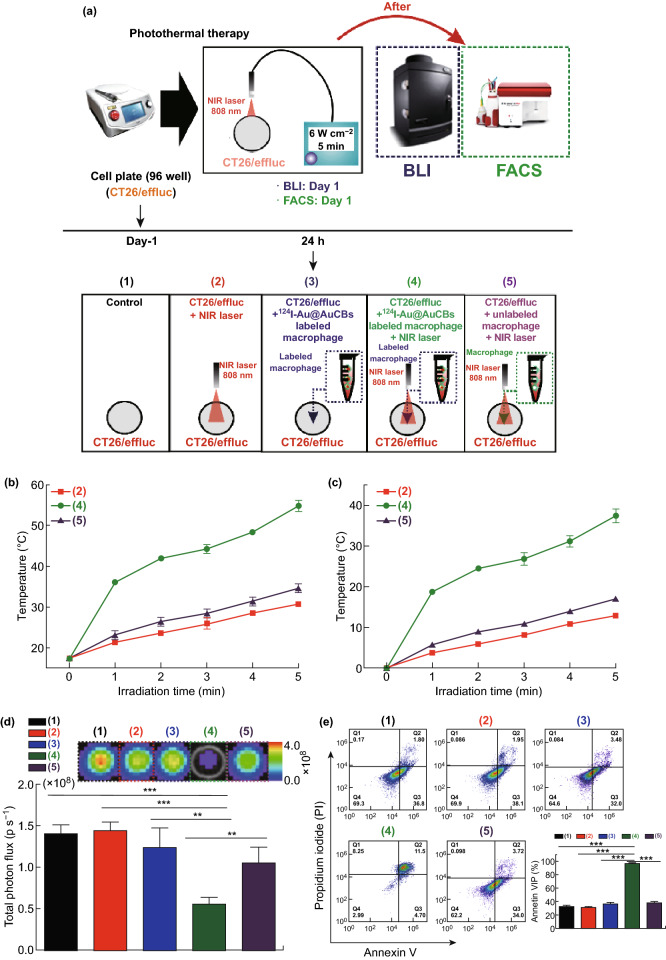
Induction of cytotoxic effects in colon cancer cells via photothermal therapy using ^124^I-Au@AuCB-labeled macrophages. **a** Scheme for ^124^I-Au@AuCB-labeled macrophage-mediated PTT in vitro. **b** Temperature and **c** temperature changes (*Δ* temperature) in unlabeled macrophages and ^124^I-Au@AuCB-labeled macrophages after irradiation with an NIR laser for 5 min. The red line indicates temperature changes in media irradiated with an NIR laser. **d**, **e** Evaluation of cell proliferation and apoptosis levels in CT26/FM alone (black), CT26/FM + NIR laser (red), CT26/FM + ^124^I-Au@AuCB-labeled macrophages (blue), CT26/FM + ^124^I-Au@AuCB-labeled macrophages + NIR laser (green), and unlabeled macrophages alone + laser (purple) using in vitro bioluminescent imaging and annexin V/PI staining, respectively. **P < 0.001, ***P < 0.0001

In the present study, ^124^I-Au@AuCBs were directly introduced into murine macrophage Raw 264.7 cells and microscopic examination was performed to confirm the presence of the particles within the macrophages. Concurrent with previous reports, ^124^I-Au@AuNPs were easily taken up by macrophages without any assistance and labeled cells were darkened owing to the presence of AuNPs (data not shown). When laser irradiation was applied to ^124^I-Au@AuNP-labeled macrophages, effective photothermal conversion was observed in ^124^I-Au@AuNP-labeled macrophages, but not in unlabeled macrophages (Fig. 5b, c). ^124^I-Au@AuNP-labeled macrophages reached temperatures greater than 43 °C after irradiation, which is essential for successful PTT.

Thereafter, we evaluated whether ^124^I-Au@AuNP-labeled macrophages resulted in the death of colon cancer cells in the following groups: control group, NIR laser group, ^124^I-Au@AuNP-labeled macrophages group, ^124^I-Au@AuNP-labeled macrophages + NIR laser treatment group, and unlabeled macrophages + NIR laser treatment group. To visualize cell viability in vitro and in vivo, we engineered CT26 cells to co-express firefly luciferase and mCherry as optical reporter genes (CT26/FM cells). Briefly, CT26/FM cells were co-incubated with ^124^I-Au@AuNP-labeled macrophages and their respective cells were irradiated. After an additional incubation for 12 h, the in vitro effects of PTT were confirmed using BLI and apoptosis analysis. In vitro BLI (Fig. 5d) and apoptosis assays (Fig. 5e) showed the lowest BLI signals and the highest number of annexin V/PI-positive cells in the ^124^I-Au@AuNP-labeled macrophages + NIR laser treatment group. These findings indicate that ^124^I-Au@AuCBs engulfed by macrophages retain the potential for photothermal conversion, thereby effectively eliminating colon cancer cells surrounding the ^124^I-Au@AuNP-labeled macrophages.

**Fig. 6:**
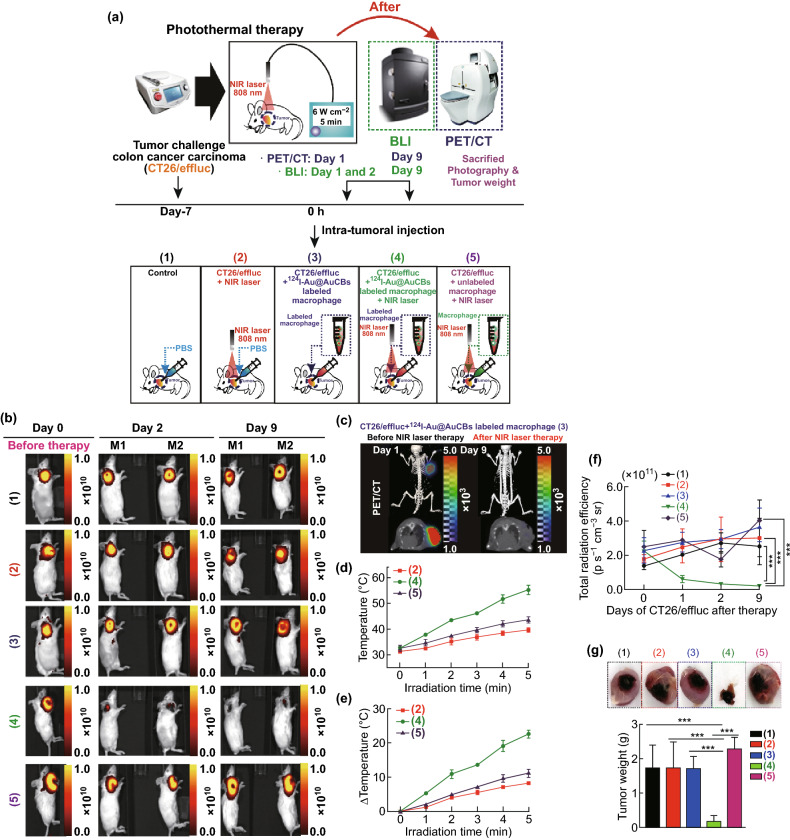
Ablation of colon cancer by ^124^I-Au@AuCB-labeled macrophage-mediated PTT. **a** Scheme for ^124^I-Au@AuCB-labeled macrophage-mediated PTT in vivo. In vivo PET/CT imaging of CT26/FM tumor-bearing mice, intratumorally injected with ^124^I-Au@AuCB-labeled macrophages. Experimental groups: (1) control, (2) laser alone, (3) ^124^I-Au@AuCB-labeled macrophages, (4) ^124^I-Au@AuCB-labeled macrophages + laser-treated, and (5) macrophages + laser-treated. **b** In vivo FLI showing the therapeutic response to ^124^I-Au@AuCB-labeled macrophage-mediated PTT. **c** PET/CT imaging of CT26/FM tumor-bearing mice administered either unlabeled or labeled macrophages and subsequently irradiated with an NIR laser (6 W cm^−2^, 5 min). **d** Temperature and **e** temperature changes (*Δ* temperature) in CT26/FM tumors, before and after irradiation with an NIR laser (6 W cm^−2^, 5 min). **f** Quantification of FLI signals in CT26/FM tumors, before and after PTT. **g** Photograph of an excised CT26/FM tumor and determination of tumor weight from respective groups. In vivo experiments were performed in duplicate (*n* = 7 mice per group). ***P < 0.0001

Finally, the in vivo effects of PTT using ^124^I-Au@AuCB-labeled macrophages were evaluated in CT26/FM tumor-bearing mice (Fig. 6a). When the tumors approached approximately 100–120 mm^3^, mice were divided into the following five groups: control, laser-treated, ^124^I-Au@AuNP-labeled macrophages, ^124^I-Au@AuNP-labeled macrophages + laser-treated, and unlabeled macrophages + laser-treated groups. PET/CT imaging was performed 5 min after the intratumoral injection of ^124^I-Au@AuNP-labeled macrophages. After image acquisition, NIR laser irradiation (6.0 W cm^−2^, 808 nm) was administered to the tumor site, 3 h after macrophage injection. Temperature changes in tumor lesions were monitored using a digital thermometer. The therapeutic effects of PTT were determined by in vivo fluorescent imaging of the mCherry reporter gene. Zhibin et al. [31] also reported the therapeutic potential of the delivery of PTT agents via macrophages. They demonstrated more effective distribution around the tumor site and could overcome the extracellular matrix barrier and penetrate deeper into tumor sites, resulting in enhanced tumor inhibition compared with intratumoral injection of the PTT agent alone. The present study differed from the study by Zhibin et al., in terms of the irradiation time points. In the previous study, tumors were irradiated 48 h after the intratumoral injection of labeled macrophages. In our study, tumors sites were directly irradiated by an NIR laser 3 h after transferring the ^124^I-Au@AuNP-labeled macrophages. Using our current approach, ^124^I-Au@AuCB-labeled macrophages were observed to be evenly distributed in tumor lesions upon PET/CT imaging, with intense signals in tumor lesions receiving ^124^I-Au@AuCB-labeled macrophages; this indicated the successful transfer of ^124^I-Au@AuCBs into tumor lesions, with macrophages acting as Trojan horses (Fig. 6c). As seen in Fig. 6d, e, tumors injected with ^124^I-Au@AuCB-labeled macrophages approached 55.2 °C within 5 min, at which point cancer cells were effectively eliminated. Consistent with this observation, fluorescent signals were markedly reduced in the ^124^I-Au@AuNP-labeled macrophages + laser-treated group at post-therapy day 1, and the signals then gradually decreased until day 9 (Fig. 6b, f). Accordingly, tumor weight was also the lowest in the macrophages + laser-treated group (Fig. 6g). However, the temperature in the tumors of the control + laser-treated and unlabeled macrophage + laser-treated groups was less than 43 °C. Accordingly, we did not observe significant tumor reduction in the control + laser-treated, ^124^I-Au@AuNP-labeled macrophages, or unlabeled macrophages + laser-treated groups. Recently, Baek et al. [22] reported that peritumoral injection of macrophages loaded with gold nanoshells is more effective than intratumoral injection for PTT, due to complete migration of the macrophages throughout the tumor. Of note, we also need to further investigate the effect of ^124^I-Au@AuNP-loaded macrophages on PTT, when they are administered via another injection route, such as peritumoral injection.

## Conclusion

This study evaluated the possibility of using ^124^I-Au@AuCBs as a novel theranostic nanoplatform for in vivo PTT. ^124^I-Au@AuCBs exhibited great photothermal conversion ability, and macrophage labeling was achieved via a simple incubation, with no associated cytotoxicity. Furthermore, the effects of ^124^I-Au@AuCBs on PTT were retained upon macrophage uptake, thereby inducing discernible cell death in tumors in vitro. More importantly, it is possible to visualize the successful delivery of ^124^I-Au@AuCBs to tumor lesions by using macrophages as Trojan horses, owing to the presence of ^124^I, which can be detected by PET imaging. Finally, ^124^I-Au@AuCB-labeled macrophages resulted in significant tumor ablation, suggesting that this is a potentially effective photothermal approach for optimizing nanoplatform delivery in vivo. Further studies are required to examine the tracking of ^124^I-Au@AuNP-loaded macrophages to tumor lesions after intravenous injection and to assess the potential for translation of our experimental findings to clinical applications in cancer therapy.

## Electronic supplementary material

Below is the link to the electronic supplementary material.

Supplementary material 1 (PDF 823 kb)

## References

[1] Boisselier E, Astruc D (2009). Gold nanoparticles in nanomedicine: preparations, imaging, diagnostics, therapies and toxicity. Chem. Soc. Rev..

[2] Hu M, Chen J, Li ZY, Au L, Hartland GV, Li X, Marquez M, Xia Y (2006). Gold nanostructures: engineering their plasmonic properties for biomedical applications. Chem. Soc. Rev..

[3] Melancon MP, Lu W, Yang Z, Zhang R, Cheng Z (2008). In vitro and in vivo targeting of hollow gold nanoshells directed at epidermal growth factor receptor for photothermal ablation therapy. Mol. Cancer Ther..

[4] Ji X, Shao R, Elliott AM, Stafford RJ, Esparza-Coss E (2007). Bifunctional gold nanoshells with a superparamagnetic iron oxide–silica core suitable for both MR imaging and photothermal therapy. J. Phys. Chem. C.

[5] Liu H, Chen D, Li L, Liu T, Tan L, Wu X, Tang F (2011). Multifunctional gold nanoshells on silica nanorattles: a platform for the combination of photothermal therapy and chemotherapy with low systemic toxicity. Angew. Chem. Int. Ed..

[6] Li Z, Huang P, Zhang X, Lin J, Yang S (2009). RGD-conjugated dendrimer-modified gold nanorods for in vivo tumor targeting and photothermal therapy. Mol. Pharm..

[7] Chen J, Wang D, Xi J, Au L, Siekkinen A (2007). Immuno gold nanocages with tailored optical properties for targeted photothermal destruction of cancer cells. Nano Lett..

[8] Chen J, Glaus C, Laforest R, Zhang Q, Yang M, Gidding M, Welch MJ, Xia Y (2010). Gold nanocages as photothermal transducers for cancer treatment. Small.

[9] Au L, Zheng D, Zhou F, Li Z-Y, Li X, Xia Y (2008). A quantitative study on the photothermal effect of immuno gold nanocages targeted to breast cancer cells. ACS Nano.

[10] Yang J, Shen D, Zhou L, Li W, Li X (2013). Spatially confined fabrication of core–shell gold nanocages@mesoporous silica for near-infrared controlled photothermal drug release. Chem. Mater..

[11] Tong L, Wei Q, Wei A, Cheng JX (2009). Gold nanorods as contrast agents for biological imaging: optical properties, surface conjugation and photothermal effects. Photochem. Photobiol..

[12] Dickerson EB, Dreaden EC, Huang X, El-Sayed IH, Chu H, Pushpanketh S, McDonald JF, El-Sayed MA (2008). Gold nanorod assisted near-infrared plasmonic photothermal therapy (pptt) of squamous cell carcinoma in mice. Cancer Lett..

[13] Alkilany AM, Thompson LB, Boulos SP, Sisco PN, Murphy CJ (2012). Gold nanorods: their potential for photothermal therapeutics and drug delivery, tempered by the complexity of their biological interactions. Adv. Drug Deliv. Rev..

[14] Tong L, Zhao Y, Huff TB, Hansen MN, Wei A, Cheng JX (2007). Gold nanorods mediate tumor cell death by compromising membrane integrity. Adv. Mater..

[15] von Maltzahn G, Park J-H, Agrawal A, Bandaru NK, Das SK, Sailor MJ, Bhatia SN (2009). Computationally guided photothermal tumor therapy using long-circulating gold nanorod antennas. Cancer Res..

[16] Lin J, Wang S, Huang P, Wang Z, Chen S (2013). Photosensitizer-loaded gold vesicles with strong plasmonic coupling effect for imaging-guided photothermal/photodynamic therapy. ACS Nano.

[17] Yuan H, Khoury CG, Wilson CM, Grant GA, Bennett AJ, Vo-Dinh T (2012). In vivo particle tracking and photothermal ablation using plasmon-resonant gold nanostars. Nanomed. Nanotechnol..

[18] Wang Y, Black KC, Luehmann H, Li W, Zhang Y (2013). Comparison study of gold nanohexapods, nanorods, and nanocages for photothermal cancer treatment. ACS Nano.

[19] Cheng L, Wang C, Feng L, Yang K, Liu Z (2014). Functional nanomaterials for phototherapies of cancer. Chem. Rev..

[20] Wang AZ, Langer R, Farokhzad OC (2012). Nanoparticle delivery of cancer drugs. Annu. Rev. Med..

[21] Yi X, Yang K, Liang C, Zhong X, Ning P (2015). Imaging-guided combined photothermal and radiotherapy to treat subcutaneous and metastatic tumors using iodine-131-doped copper sulfide nanoparticles. Adv. Funct. Mater..

[22] Yang TD, Choi W, Yoon TH, Lee KJ, Lee J-S (2016). In vivo photothermal treatment by the peritumoral injection of macrophages loaded with gold nanoshells. Biomed. Opt. Express.

[23] Wang B-K, Yu X-F, Wang J-H, Li Z-B, Li P-H, Wang H, Song L, Chu PK, Li C (2016). Gold-nanorods-siRNA nanoplex for improved photothermal therapy by gene silencing. Biomaterials.

[24] Liu Y, Yang M, Zhang J, Zhi X, Li C, Zhang C, Pan F, Wang K, Yang Y, Martinez de la Fuentea J (2016). Human induced pluripotent stem cells for tumor targeted delivery of gold nanorods and enhanced photothermal therapy. ACS Nano.

[25] Tang S, Chen M, Zheng N (2014). Sub-10-nm Pd nanosheets with renal clearance for efficient near-infrared photothermal cancer therapy. Small.

[26] Kang S, Bhang SH, Hwang S, Yoon JK, Song J, Jang HK, Kim S, Kim BS (2015). Mesenchymal stem cells aggregate and deliver gold nanoparticles to tumors for photothermal therapy. ACS Nano.

[27] Choi M-R, Stanton-Maxey KJ, Stanley JK, Levin CS (2007). A cellular trojan horse for delivery of therapeutic nanoparticles into tumors. Nano Lett..

[28] Madsen SJ, Baek S-K, Makkouk AR, Krasieva T, Hirschberg H (2012). Macrophages as cell-based delivery systems for nanoshells in photothermal therapy. Annu. Biomed. Eng..

[29] Choi J, Kim H-Y, Ju EJ, Jung J, Park J (2012). Use of macrophages to deliver therapeutic and imaging contrast agents to tumors. Biomaterials.

[30] Stephan MT, Stephan SB, Bak P, Chen J, Irvine DJ (2012). Synapse-directed delivery of immunomodulators using t-cell-conjugated nanoparticles. Biomaterials.

[31] Li Z, Huang H, Tang S, Li Y, Yu X-F (2016). Small gold nanorods laden macrophages for enhanced tumor coverage in photothermal therapy. Biomaterials.

[32] Lee SB, Kumar D, Li Y, Lee I-K, Cho SJ (2018). Pegylated crushed gold shell-radiolabeled core nanoballs for in vivo tumor imaging with dual positron emission tomography and cerenkov luminescent imaging. J. Nanobiotechnol..

[33] Lee SB, Lee S-W, Jeong SY, Yoon G, Cho SJ (2017). Engineering of radioiodine-labeled gold core–shell nanoparticles as efficient nuclear medicine imaging agents for trafficking of dendritic cells. ACS Appl. Mater. Interfaces.

[34] Cheng X, Tian X, Wu A, Li J, Tian J (2015). Protein corona influences cellular uptake of gold nanoparticles by phagocytic and nonphagocytic cells in a size-dependent manner. ACS Appl. Mater. Interfaces.

[35] Aderem A, Underhill DM (1999). Mechanisms of phagocytosis in macrophages. Annu. Rev. Immunol..

[36] Irvine DJ, Hanson MC, Rakhra K, Tokatlian T (2015). Synthetic nanoparticles for vaccines and immunotherapy. Chem. Rev..

[37] Anselmo AC, Mitragotri S (2014). Cell-mediated delivery of nanoparticles: taking advantage of circulatory cells to target nanoparticles. J. Control Release.

