# Supplementary material for: Crushed Gold Shell Nanoparticles Labeled with Radioactive Iodine as a Theranostic Nanoplatform for Macrophage-Mediated Photothermal Therapy
Source: Nanomicro Lett. 2019 May 3;11:36. doi: 10.1007/s40820-019-0266-0 (PMC7770679; doi:10.1007/s40820-019-0266-0)
Supplement: Supplementary file 1 — Supplementary material 1 (PDF 823 kb) [file 40820_2019_266_MOESM1_ESM.pdf]

Supporting Information for

Crushed Gold Shell Nanoparticles Labeled with Radioactive Iodine as a Theranostic Nanoplatform for Macrophage-Mediated Photothermal Therapy

Sang Bong Lee[1], Jae-Eon Lee[2], Sung Jin Cho[1, 3], Jungwook Chin[1], Sang Kyoon Kim[7], In-Kyu Lee[3, 5], Sang-Woo Lee[3, 4], Jaetae Lee[4, 6], Yong Hyun Jeon[3, 7, *]

[1]New Drug Development Center, Daegu-Gyeongbuk Medical Innovation Foundation, Daegu, South Korea

[2]Department of Biomaterials Science, College of Natural Resources and Life Science/Life and Industry Convergence Research Institute, Pusan National University, Pusan, South Korea

[3]Leading-edge Research Center for Drug Discovery and Development for Diabetes and Metabolic Disease, Kyungpook National University Hospital, Daegu, 702-210, South Korea

[4]Department of Nuclear Medicine, Kyungpook National University Hospital, Daegu, 702-210, South Korea

[5]Department of Internal Medicine, Kyungpook National University School of Medicine, Daegu 700-721, South Korea

[6]Daegu-Gyeongbuk Medical Innovation Foundation, Daegu 360-4, South Korea

[7]Laboratory Animal Center, Daegu-Gyeongbuk Medical Innovation Foundation, Daegu 360-4, South Korea

Sang Bong Lee and Jae-Eon Lee contributed equally to this study.

*Corresponding author. E-mail: jeon9014@gmail.com (Yong Hyun Jeon)

Supplementary Figures

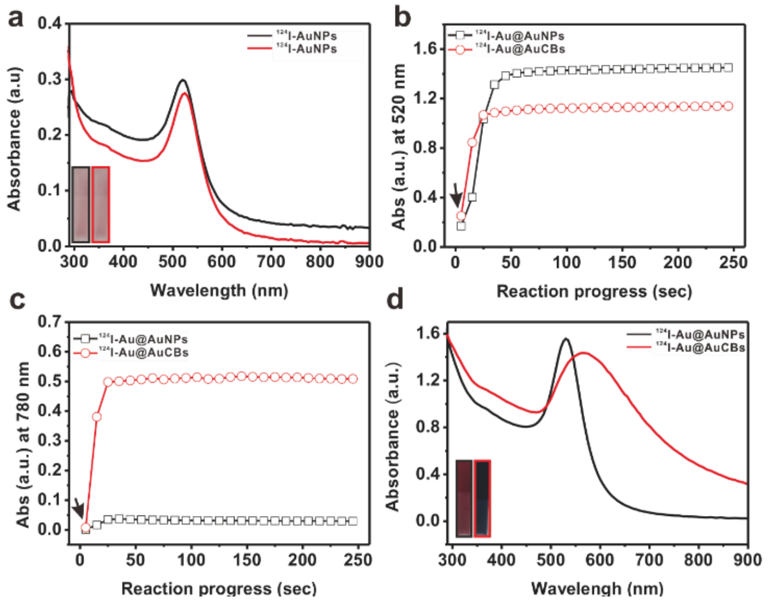

Fig. S1 Characterization of ^{124}I-Au@AuNPs and ^{124}I-Au@AuCBs. a Normalized absorption spectrum of ^{124}I-AuNPs before Au shell formation. **b, c** The reaction rate of Au-shell coverage on ^{124}I-Au@AuNPs at 520 and 600 nm. **d** Normalized absorption spectra of ^{124}I-Au@AuNPs and ^{124}I-Au@AuCBs

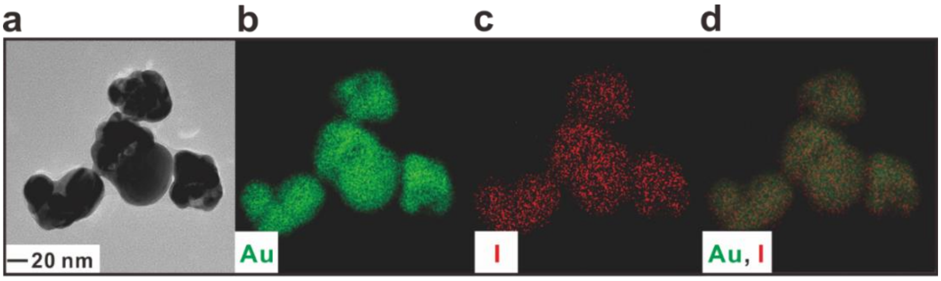

Fig. S2 a TEM micrograph of ^{124}I-Au@AuCBs and energy dispersive X-ray-based elemental mapping of **b** Au and **c** I. A merged image is also shown **d**.

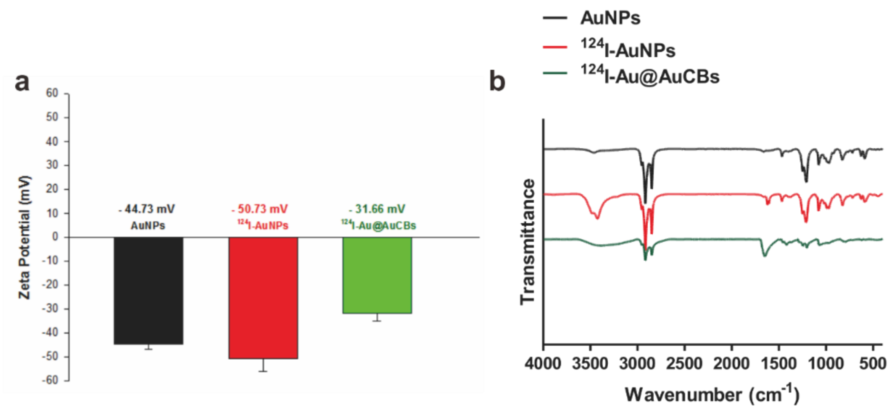

Fig. S3 a Zeta-potentials, **b** FT-IR of AuNPs, ^{124}I-AuNPs, and ^{124}I-Au@AuCBs

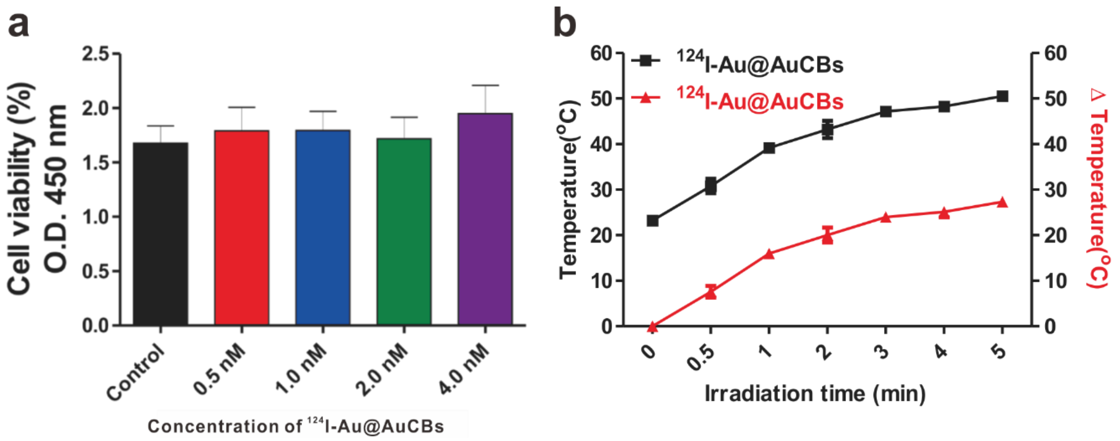

Fig. S4 Toxicity of ^{124}I-Au@AuCBs in colon cancer CT26/cells. a Cells were seeded in 96-well plates and treated with ^{124}I-Au@AuCBs for 24 h. Cell proliferation was analyzed using the Cell Counting Kit-8 (CCK-8) assay. **b** Curves showing changes in the temperature (Δ) of the ^{124}I-Au@AuCBs upon irradiation of the colon cancer CT26 cells with an NIR laser for 5 min

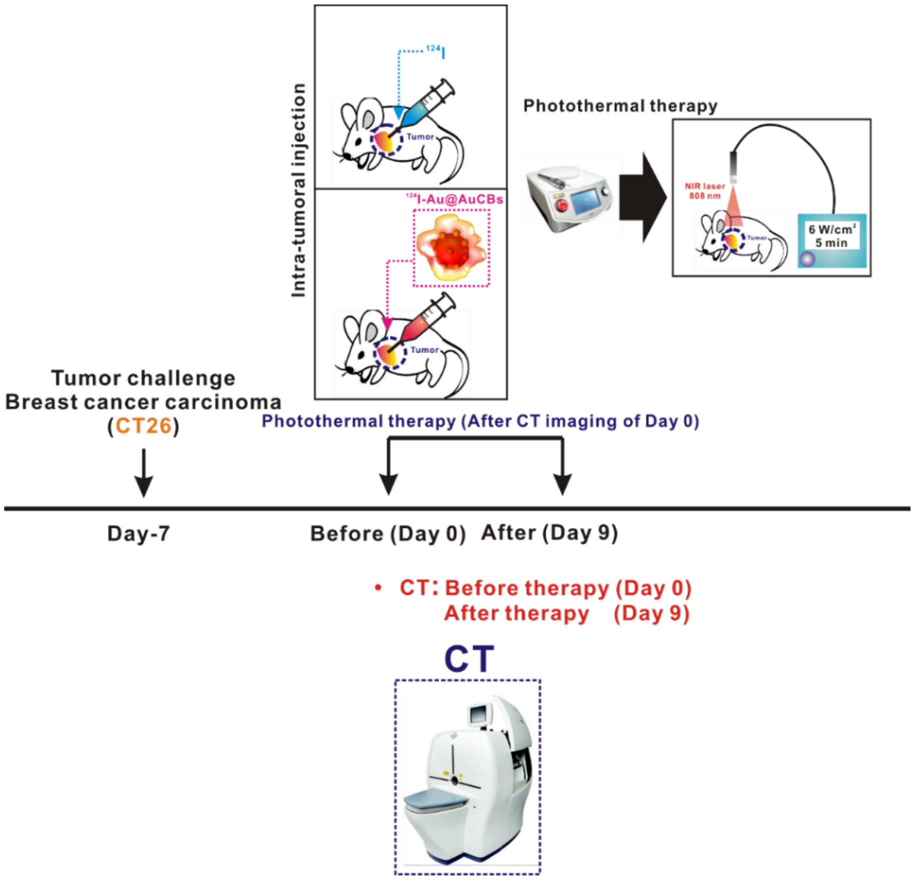

Fig. S5 Schematic representations of the protocol for *in vivo* photothermal therapy using ^{124}I-Au@AuCBs for CT26 tumor-bearing mice. CT26 tumor-bearing mice received free ^{124}I and ^{124}I-Au@AuCBs via intratumoral injection. Before and after therapy, changes in tumor volume were monitored post-photothermal therapy via positron emission tomography/computed tomography at indicated time points. *In vivo* experiments were performed in duplicate (n=7 mice per group)
